# How do we get from hyperexcitability to excitotoxicity in amyotrophic lateral sclerosis?

**DOI:** 10.1093/brain/awae039

**Published:** 2024-02-26

**Authors:** G Lorenzo Odierna, Steve Vucic, Marcus Dyer, Tracey Dickson, Adele Woodhouse, Catherine Blizzard

**Affiliations:** Tasmanian School of Medicine, University of Tasmania, Hobart, TAS 7000, Australia; Brain and Nerve Research Center, The University of Sydney, Sydney 2050, Australia; Menzies Institute for Medical Research, University of Tasmania, Hobart, TAS 7000, Australia; Department of Pharmaceutical and Pharmacological Sciences, Center for Neurosciences, Vrije Universiteit Brussel (VUB), 1090 Brussels, Belgium; Menzies Institute for Medical Research, University of Tasmania, Hobart, TAS 7000, Australia; The Wicking Dementia Centre, University of Tasmania, Hobart, TAS 7000, Australia; Tasmanian School of Medicine, University of Tasmania, Hobart, TAS 7000, Australia; Menzies Institute for Medical Research, University of Tasmania, Hobart, TAS 7000, Australia

**Keywords:** amyotrophic lateral sclerosis, hyperexcitability, excitotoxicity, dying forward, homeostasis

## Abstract

Amyotrophic lateral sclerosis is a devastating neurodegenerative disease that, at present, has no effective cure. Evidence of increased circulating glutamate and hyperexcitability of the motor cortex in patients with amyotrophic lateral sclerosis have provided an empirical support base for the ‘dying forward’ excitotoxicity hypothesis. The hypothesis postulates that increased activation of upper motor neurons spreads pathology to lower motor neurons in the spinal cord in the form of excessive glutamate release, which triggers excitotoxic processes. Many clinical trials have focused on therapies that target excitotoxicity via dampening neuronal activation, but not all are effective. As such, there is a growing tension between the rising tide of evidence for the ‘dying forward’ excitotoxicity hypothesis and the failure of therapies that target neuronal activation.

One possible solution to these contradictory outcomes is that our interpretation of the current evidence requires revision in the context of appreciating the complexity of the nervous system and the limitations of the neurobiological assays we use to study it. In this review we provide an evaluation of evidence relevant to the ‘dying forward’ excitotoxicity hypothesis and by doing so, identify key gaps in our knowledge that need to be addressed. We hope to provide a road map from hyperexcitability to excitotoxicity so that we can better develop therapies for patients suffering from amyotrophic lateral sclerosis.

We conclude that studies of upper motor neuron activity and their synaptic output will play a decisive role in the future of amyotrophic lateral sclerosis therapy.

## Teaser

Odierna *et al.* evaluate preclinical and clinical evidence for brain-initiated toxicity in amyotrophic lateral sclerosis, and identify key roadblocks and gaps in understanding that must be tackled in order to develop effective therapies.

## Introduction

It has become increasingly evident that a key pathophysiological event in the progression of amyotrophic lateral sclerosis is the induction of hyperexcitability of the motor cortex.^1^ Studies of patients with amyotrophic lateral sclerosis have converged upon the conclusion that early motor cortex hyperexcitability, as defined by measures of cortical network function (such as reduced short intracortical inhibition and increased motor evoked potential amplitude), is a pathogenic feature.^2-4^ Such findings have significantly contributed to our understanding of this rapidly progressing neurodegenerative disease. They have also strengthened support for the ‘dying forward’ hypothesis, which traditionally describes pathological spread as a feed-forward process that relies on glutamate excitotoxicity (see Shaw and Ince^5^ and King *et al*.^6^ for more detailed reviews). Much attention has been turned towards the development of therapies that aim to suppress activation of neurons by glutamate as a treatment for amyotrophic lateral sclerosis. However, apart from riluzole, most of these treatments have failed to extend patient lifespans or effectively treat their symptoms.^7-12^ Despite frequent glimmers of hope, the amyotrophic lateral sclerosis research field has a history of therapeutic trails targeting excitotoxicity that fail to reach efficacy, beginning with lamotrigine and dextromethorphan close to 30 years ago.^13-15^ There are many possible explanations for why the efficacy of new treatments that target glutamate-mediated hyperexcitability are not consistently effective, such as the long delay between presentation and diagnosis (typically 10–16 months from symptom onset),^16^ a lack of effective biomarkers,^17^ or the overwhelming heterogeneity of the disease.^18,19^ One conclusion that needs to be considered is that our current hypotheses about the mechanisms that take a hyperexcitable motor cortex to excitotoxicity and neuronal degeneration in amyotrophic lateral sclerosis need to be re-examined. An incomplete understanding of disease mechanisms can result in targeting incorrect pathways, or inadvertently suppressing homeostatic processes that have been induced to counteract a neuronal dysfunction. A recent example of this came from a randomized phase 2 clinical trial for perampanel, an AMPA receptor antagonist used to treat epilepsy, as a therapy for patients with sporadic amyotrophic lateral sclerosis. Daily administration of perampanel worsened patients’ functional rating scores and increased the frequency of adverse events.^20^ Collectively, these studies provide the impetus needed to consider broadening interpretations of the role hyperexcitability plays in the disease.

The aim of the present review is to reassess recent preclinical research to identify exactly what is missing with respect to the ‘dying forward’ excitotoxicity hypothesis so that we can better position ourselves to develop effective therapies. We aim to evaluate interpretations of clinical and preclinical pathophysiological amyotrophic lateral sclerosis studies with a specific focus on excitability, activity and neurotransmission.

With this in mind, it is important to define some core terminology. ‘Excitability’ broadly refers to input-output relationships and can be used to describe entire networks or individual neurons, depending on the assay being used. In the context of networks, it refers to the propensity to produce a measurable functional output of the network in question in response to spatially broad stimulation (e.g. the motor evoked potential following stimulation of the motor cortex). In the context of neurons, it refers to the propensity to produce action potentials in response to depolarizing currents. ‘Activity’ is a separate term from ‘excitability’ and instead refers to the aggregate output of a network or a neuron over time. This can refer to resting levels of activity that occur spontaneously or the activity profile that occurs during recruitment of networks or neurons in their relevant context (e.g. the pattern of lower motor neuron activation during performance of a motor task). It is important to recognize that excitability alone is not predictive of activity and that for the ‘dying forward’ hypothesis, it is activity that is ultimately of interest.

### Defining ‘dying forward’ spread of pathology and key evidence for the excitability hypothesis

Although ‘excitotoxicity’ and ‘dying forward’ are often conflated in amyotrophic lateral sclerosis research, they are technically not mutually inclusive ideas. Excitotoxicity classically refers to abnormal acute or chronic activation of intracellular calcium signalling cascades via glutamate receptor-dependent pathways.^21^ It is a process that is strongly associated with demise of neurons via either apoptotic or necroptotic cell death.^22,23^ The dying forward hypothesis highlights the primacy of the motor cortex in the initiation and propagation of degenerative pathways that converge onto lower motor neurons, leading to neurodegeneration and the amyotrophic lateral sclerosis phenotype.^24^ The most widely recognized form of the ‘dying forward’ hypothesis is one that integrates the idea of excitotoxicity as the physiological instrument that propagates pathology from projection neurons in the motor cortex to lower motor neurons in the spinal cord. Empirical support for this hypothesis started with the occurrence of increased CSF glutamate levels in patients with amyotrophic lateral sclerosis and was later supported by observations of motor cortex hyperexcitability. These results provide insightful evidence that the nervous system in amyotrophic lateral sclerosis patients functions differently at a very fundamental level and as such, have shaped the way we think about pathological mechanisms.

The earliest evidence for excitotoxicity in amyotrophic lateral sclerosis came from the striking similarities between the cellular pathology induced by excitotoxins and the observed pathological features in post-mortem tissue of patients.^25^ Of particular interest were axonal, dendritic and somatic swellings seen throughout the spinal cord of amyotrophic lateral sclerosis patients,^25^ which were highly reminiscent of those seen in rodents treated with glutamate or kainic acid.^26,27^ Studies of amino acid levels in plasma and CSF supported this connection by finding that patients with amyotrophic lateral sclerosis had high concentrations of circulating glutamate.^28-32^ The most common interpretation of these findings is that the increased circulating glutamate is a by-product of underlying neuronal hyperactivity, and that excess glutamate likely drives the excitotoxic processes. Critical evaluation of raised glutamate levels in patients, however, indicates that our understanding of the role of glutamate in amyotrophic lateral sclerosis remains incomplete. The initial findings of raised glutamate levels were not found in all patient subgroups; some studies claimed that patients had high plasma or CSF glutamate concentrations,^28-32^ whereas others found that glutamate levels were comparable to controls.^33,34^ Later studies provided some *ad hoc* clarification by identifying that high circulating glutamate in patients with amyotrophic lateral sclerosis is specifically associated with spinal onset.^35-37^ Importantly, many of the original studies reported a striking depletion of glutamate levels in brain and spinal cord tissue of patients^31,38-42^ but this was never meaningfully incorporated into later hypotheses of the disease mechanisms that focused on excitotoxicity. The reports of glutamate depletion, specifically in the ventral horn of the spinal cord, indicate the existence of a yet unrecognized degree of complexity of amyotrophic lateral sclerosis that challenges hypotheses that rely on excitotoxicity.

The ‘dying forward’ hypothesis is supported by early hyperexcitability that is observed in cortex of patients with amyotrophic lateral sclerosis.^3,4,43-45^ Cortical network excitability of patients has been explored in detail by clinical studies that have leveraged transcranial magnetic stimulation (TMS) technology alongside the accessibility of measuring corticomotor system output in the periphery. TMS uses brief pulses of electricity through a coil to generate a current that can penetrate the intact scalp and stimulate the motor cortex and all the microcircuitry within.^46^ By doing so, TMS elicits a response at muscles, which is measurable via EMG. Comparing the minimum required current to elicit an EMG response can produce an approximation of how excitable the motor cortex is. Clinical TMS studies indicate that the motor cortex in amyotrophic lateral sclerosis patients is hyperexcitable.^2-4^ This change is detectable before onset of motor symptoms in individuals with familial amyotrophic lateral sclerosis^44^ and before manifestation of measurable changes in peripheral nerve evoked responses.^2^ Cortical hyperexcitability is one of the most reliable biosignatures of amyotrophic lateral sclerosis and can differentiate the disease from its mimics.^47-50^ However, how cortical excitability and neurodegeneration in amyotrophic lateral sclerosis mechanistically relate to each other remains to be fully elucidated.

**Figure 1:**
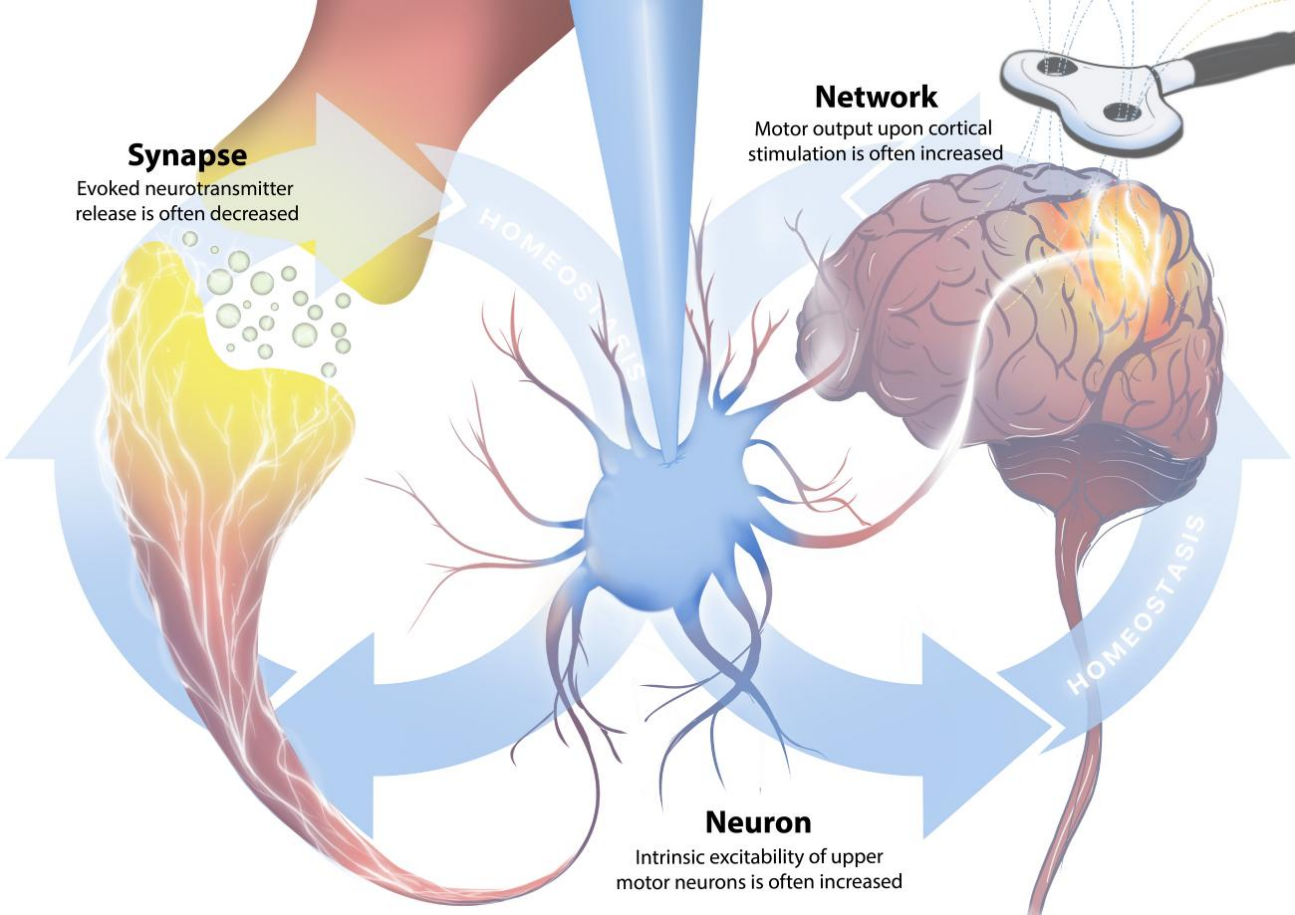
**The complexity of the corticomotor system and the limitations of the tools we use to study it**. Amyotrophic lateral sclerosis has been studied at three levels across the nervous system: networks, neurons and synapses. **Network**: Our understanding of network physiology has been predominantly derived from research implementing transcranial magnetic stimulation (TMS) to patients with amyotrophic lateral sclerosis. TMS can induce activation of brain regions to measure the excitability of networks (glowing brain region under the coil). Clinical TMS studies have revealed that the cortex of patients is often more excitable, such that peripheral motor responses can be more easily induced for any intensity of stimulus provided to the motor region. TMS is limited by cellular resolution and only comments on excitability. As such, the endogenous activity of motor networks, and the cells within them, in patients remains unknown. **Neuron**: Our understanding of neuronal physiology in amyotrophic lateral sclerosis has been predominantly derived from research implementing whole-cell patch-clamp of neurons in the corticomotor system of preclinical rodent models. Patch-clamp can measure currents that underlie neuronal function, but this is limited to the region of neurons that can be confidently controlled by the experimenter (blue region of the neuron depicts area of a neuron that is likely clamped by the pipette and accurately recorded from). Patch-clamp studies have revealed that layer 5 projection neurons of the motor cortex are often intrinsically hyperexcitable, such that their likelihood of firing action potentials is increased for any intensity of injected current. Patch-clamp is limited by its poor synaptic resolution, can only measure single neurons in isolation and only comments on excitability. As such, the endogenous activity of upper motor neurons remains unknown. **Synapse**: Our understanding of synaptic physiology in amyotrophic lateral sclerosis has been predominantly derived from research implementing intracellular recordings of muscle end plate potentials. End plate potential recordings measure properties of synaptic neurotransmission but are biased towards lower motor neurons and neuromuscular physiology. End plate potential recordings across multiple different model organism species have revealed that evoked neurotransmitter release is often decreased in amyotrophic lateral sclerosis. There currently are no assays to measure neurotransmission properties of central neurons of the corticomotor system. As such, basal neurotransmitter release from axons of upper motor neurons remains unknown. Network-, neuron- and synapse-level changes must always be considered in the context of homeostatic adaptions, which occur to stabilize outputs based on global and local set points.

The difficulty in forming causal links between glutamate, cortical hyperexcitability and neurodegeneration in amyotrophic lateral sclerosis arises from the complexity of the corticomotor system and the limitations of the tools we use to study it. Clinical studies provide directly relevant and diagnostically rich information but must be tempered by patient care. Hence, animal models play a vital role in advancing our understanding of pathogenesis in amyotrophic lateral sclerosis. They permit for targeted interrogation of genetic, biochemical and physiological processes and thus provide an irreplaceable platform to test the validity of hypotheses (such as the dying forward hypothesis) at high mechanistic resolution. Despite this, different neurobiological assays are not definitive and only provide limited windows through which networks or brain cell function can be assessed. With all this in mind, we will descend in scales of complexity from networks to synapses (Fig. 1) to identify the strengths and limitations of the evidence each level can provide and hopefully build a road map to get from hyperexcitability to excitotoxicity.

## Networks

### Bridging the gap from excitability to activity: cortical network calcium imaging in mice

Studies using preclinical animal models of amyotrophic lateral sclerosis allow for interrogation of network and/or neuron physiology at high spatiotemporal resolution and broadly across brain regions. Recent studies investigating neuronal activity using *in vivo* calcium imaging in small animal models of amyotrophic lateral sclerosis have begun to bridge the gap between observations of network hyperexcitability and what this means for network activity. The importance of exploring network activity cannot be understated because it holds the potential to inform the development of meaningful activity-modulating therapies for patients. One study on mice harbouring expansion repeats of C9orf72 (Table 1) applied wireless photometry to indirectly measure the compound activity of corticospinal projection neurons via GCaMP6f signals from their axonal projections in white matter tracts. Despite finding a marked loss of neurons in the motor cortex, the authors reported that the output from those that remained was enhanced during high-speed locomotion, implying increased endogenous task-specific output.^64^ This kind of increased neuronal activity has also been observed in a study of a nuclear localization sequence-deficient fused in sarcoma mouse model (FUS^ΔNLS^, Table 1), which assessed spontaneous GCaMP6s signals of layer 2/3 motor cortex neurons in anaesthetized mice via cranial window.^65^ Although neither of these studies measured whole networks or directly assessed corticospinal projection neurons, they both provide tantalizing hints that network activity might skew towards hyperactivity in amyotrophic lateral sclerosis. Contrasting these studies, assessment of neuronal activity of layer 2/3 motor cortex neurons of mice that overexpress human superoxide dismutase 1 harbouring the G93A mutation (SOD1^G93A^, Table 1) found that neuronal activity is indistinguishable from controls and the authors concluded that network activity in amyotrophic lateral sclerosis may be stabilized by strong homeostatic adaptations.^66^ Given that this work was performed at a late symptomatic stage, it raises the possibility that there is a complex progression of destabilization and stabilization of network activity over the time course of the disease. In support of this idea, a study that applied *in vivo* calcium imaging to awake mice found that conditional knock out of transactive response DNA-binding protein 43 kDa (TDP-43, Table 1) resulted in hyperactivity of prefrontal cortex neurons, followed by silencing and then cellular death.^67^ Although this assessment was not performed in the motor cortex, it demonstrates that the network dysfunction can manifest as increased or decreased activity depending on the stage of disease.

**Table 1:** Genes and mutations used to model amyotrophic lateral sclerosis in mice discussed in this review article

Gene	Function	Insult used	Description
Superoxide dismutase 1 (SOD1)	Free radical scavenging	SOD1^G93A^	Mutations in SOD1 are strongly associated with development of familial amyotrophic lateral sclerosis.^51^ The SOD1^G93A^ mouse overexpresses human SOD1 harbouring the disease-linked G93A amino acid substitution mutation. Expression is driven off the human SOD1 promoter.^52^
Transactive response DNA binding protein 43 kDA (TDP-43)	DNA and RNA metabolism	TDP-43^A315T^	TDP-43 is a major component of ubiquitinated cytoplasmic inclusions found ubiquitously in amyotrophic lateral sclerosis patients.^53^ Mutations in TDP-43 are also associated with development of amyotrophic lateral sclerosis.^54^ The TDP-43^A315T^ mouse overexpresses human TDP-43 harbouring the disease-linked A315T amino acid substitution mutation. Expression is driven off the murine prion protein (PrP) promoter.^55^
		TDP-43^Q331K^	The TDP-43^Q331K^ mouse overexpresses human TDP-43 harbouring the disease-linked Q331K amino acid substitution mutation. Expression is driven off the murine PrP promoter.^56^
		TDP-43^ΔNLS^	The TDP-43^ΔNLS^ mouse model is based on the doxycycline-inducible gene expression system. It uses a promoter-specific tetracycline transactivator to direct cell type-specific overexpression of human TDP-43 with an ablated nuclear localization sequence (NLS).^57^
Fused in sarcoma (FUS)	DNA and RNA metabolism	FUS^ΔNLS^	Mutations in FUS are associated with familial amyotrophic lateral sclerosis patients.^58^ Disease-linked mutant FUS proteins preferentially associate with cytoplasmic stress granules.^59^ The FUS^ΔNLS^ mouse features engineering of the endogenous mouse *Fus* gene to incorporate a premature STOP codon, resulting in a C-terminally truncated NLS-deficient protein that accumulates in the cytoplasm.^60^
Chromosome 9 open reading frame 72 (C9orf72)	Autophagy and vesicle trafficking	C9-500	Intronic GGGGCC (G_4_C_2_) repeats are typically found within the *C9orf72* gene between exon 1a and exon 1b.^61^ In healthy controls, the repeats occur <30 times^62^ but in some amyotrophic lateral sclerosis patients, the sequence repeats can be expanded up to hundreds of times.^61^ The C9-500 mouse harbours ∼500 G_4_C_2_ expansion repeats.^63^

The importance of these findings lies in the distinction between excitability and activity. Despite the many studies that have demonstrated motor cortical network hyperexcitability in amyotrophic lateral sclerosis, the activity status of networks along the corticomotor system remains a mystery. This is a significant obstacle to our development of effective therapies; if the network is not hyperactive then excitability-targeting treatments are unlikely to provide therapeutic benefits to patients. As such, additional work is needed to more completely assess how activity of the corticomotor network is affected over time in amyotrophic lateral sclerosis. In moving forward, it is important to recognize that all studies of networks *in vivo* suffer from the limitation that they are multi-faceted measures (Fig. 1). They are subject to many factors that complicate deciphering mechanistic information including (but likely not limited to) neuronal population composition, network connectomes, neuronal activation properties, cell intrinsic electrophysiological factors, synaptic inputs, synaptic strength, neuromodulation, brain states, glial biology and ephaptic factors. This makes it difficult to understand how the altered network excitability or activity manifests and, consequently, what kinds of therapeutics need to be designed to treat patients’ needs. As such, each part of the corticomotor system needs to be considered at the level of individual neurons so that we can understand how the insults that drive pathology of amyotrophic lateral sclerosis affects their function.

## Neurons

### Patch-clamp and interrogation of intrinsic upper and lower motor neuron excitability

A core change that must manifest to satisfy the ‘dying forward’ hypothesis is increased output of corticospinal projection neurons (hereon referred to as ‘upper motor neurons’). These neurons represent the main conduit through which information processed in the cortex is relayed to the spinal cord and as such, their role in propagating pathophysiology is fundamental. Neuronal excitbility is the product of intrinsic and extrinsic factors. Intrinsic properties of corticospinal projection neurons have been investigated extensively in amyotrophic lateral sclerosis mouse models using whole-cell patch-clamp, which involves invasively creating a tight seal between a pipette and a neuron’s membrane. This connection is used to inject current to gain insight into the electrophysiological properties of neurons, most notably intrinsic excitability. This method enables the excitability of neurons to be resolved at the expense of being able to measure activity over time (days to weeks) or circuit activity (Fig. 1). The intrinsic excitability of a neuron is determined by the types, density, distribution and kinetics of voltage-gated ion channels in the membrane. In brain slices of mice overexpressing the familial disease associated SOD1^G93A^ (Table 1), intrinsic excitability of layer 5 pyramidal neurons of the motor cortex (of which upper motor neurons are a subset) has been shown to be either increased or decreased, implying a complex progression of excitability phenotypes. Intrinsic hyperexcitability of layer 5 pyramidal neurons of the motor cortex in SOD1^G93A^ mice occurs early in postnatal developmental stages.^66,68^ Early intrinsic hyperexcitability of these neurons has also been observed in TDP-43 mouse models of amyotrophic lateral sclerosis, including overexpression of the disease-linked mutant TDP-43^A315T^ and TDP-43^ΔNLS^ (Table 1), which accumulates human TDP-43 in the cytoplasm.^69,70^ The observation of a common layer 5 pyramidal neuron hyperexcitability phenotype in rodent models suggests that increased neuronal excitability may represent a conserved pathological feature of amyotrophic lateral sclerosis. In support of this, human induced pluripotent stem cell-derived neurons also display hyperexcitability *in vitro*.^71^ Although these neurons do not have a strict upper motor neuron identity, they provide confidence that the hyperexcitability observed in rodents is meaningful to human disease.

The mechanism of increased upper motor neuron neuronal excitability has been attributed to a range of ion channels and ionic currents. Increased persistent sodium channel currents, in particular, have been observed in layer 5 pyramidal neurons of the motor cortex in SOD1^G93A^ mice and this was associated with increased contribution of Na_v_1.6 sodium channels.^68^ Increased intrinsic excitability of layer 5 pyramidal neurons has separately been linked to an altered balance between hyperpolarization-activated cyclic nucleotide gated (HCN)- and K_v_7 potassium channel (also known as KCNQ)-mediated currents in *ex vivo* brain slices of SOD1^G93A^ mice.^72^ Different sets of voltage-gated potassium, sodium and calcium channels have been reported to be up- and downregulated in corticocortical and corticospinal layer 5 pyramidal neurons.^66^ This is perhaps not surprising given that the expression of ion channels is variably co-regulated in different subtypes of neurons to establish and maintain functional outputs.^73,74^ It is important to note that multiple adaptational configurations are possible to maintain functional output at the neuron, network and behavioural levels. For instance, network level output can be maintained despite variations in the roles of neurons within the network, and behavioural output can be maintained using multiple network configurations.^74^

The excitability of lower motor neurons has been assessed in detail in rodent models of amyotrophic lateral sclerosis. The earliest investigation into lower motor neuron excitability was performed on SOD1^G93A^ E17.5 embryos.^75^ The study found that embryonic SOD1^G93A^ lower motor neurons were hyperexcitable, as measured by higher input resistance, lower rheobase and reduced dendritic arbour. Much more work has been performed on neonatal and presymptomatic mice, many of which report seemingly contradictory findings. Some studies claim that lower motor neurons display signs of hypoexcitability,^76-78^ whereas others show that lower motor neurons are instead hyperexcitable.^79-82^ Although it is possible that these discrepancies arise due to the use of different backgrounds or models (such as the low copy number SOD1^G93A^ or the SOD1^G85R^ versus the high copy number SOD1^G93A^), a more intriguing possibility is that the differing reports arise due to a combination of two factors: (i) that the effects of mutant transgenes on lower motor neuron excitability is subtype-dependent; and (ii) that the excitability profile of lower motor neurons may change over time as the disease progresses. In support of these points, most studies that segregate lower motor neurons into subtypes based on electrophysiological or molecular markers of motor units find subtype-specific responses. Martinez-Silva *et al*.^77^ found that fast fatigable lower motor neurons, which are the most vulnerable to degeneration in amyotrophic lateral sclerosis, were more likely to display signs of hypoexcitability in SOD1^G93A^ mice, whereas slow lower motor neurons appeared unaffected. Conversely, Leroy *et al*.^82^ reported that motor neurons with a delayed response to current injection were not affected, whereas those with an immediate response became hyperexcitable, a change that was attributable in part to lower rheobase and a reduction in dendritic arbour. Venugopal *et al*.^83^ investigated ocular motor neurons, which are spared in amyotrophic lateral sclerosis, and compared their responses to different types of trigeminal motor neurons. Their results suggested that fast fatigable trigeminal motor neurons exhibit hyperexcitability, slow fatigable trigeminal motor neurons exhibit hypoexcitability, and ocular motor neurons are unaffected in SOD1^G93A^ mice. As for timelines of change, Quinlan *et al*.^84^ performed a longitudinal study on the evolution of excitability changes of lower motor neurons in SOD1^G93A^ mice from P0 to 12. The authors found that although the transgenic mice did have hyperexcitable lower motor neurons, this could be attributed to an acceleration of developmental processes that drive increased excitability of these neurons.

Together, these data indicate that lower motor neurons undergo complex changes in excitability in amyotrophic lateral sclerosis. Whether these changes exacerbate or protect them from excitotoxic damage remains unclear. An interesting question that emerges from these studies is what the effects of restricted expression of amyotrophic lateral sclerosis-related transgenes only in lower motor neurons would be. Are the observed changes in excitability a physiological, adaptive response to descending pathology or do they arise from intrinsic molecular processes within the lower motor neurons following gain- or loss-of-function of proteins such as SOD1 or TDP-43? Further, how would the motor cortex change in response to focal expression of transgenes only in lower motor neurons? More work will be needed to answer these questions.

### Presynaptic input onto upper motor neurons and the excitability of cortical interneurons

Many factors influence neuronal inputs including the number of excitatory and inhibitory synapses, the frequency, strength and timing of synaptic currents mediated by the activity of presynaptic neurons. Electrophysiological measurements of excitatory and inhibitory post-synaptic currents (EPSCs and IPSCs, respectively) have been explored in corticospinal projection neurons using patch-clamp techniques and have been used to draw conclusions about synaptic inputs. Postsynaptic currents display marked changes that follow a pattern along the progression of disease. Increased EPSC frequency has been observed in layer 5 pyramidal neurons of the motor cortex before symptom onset in SOD1^G93A^ and TDP-43^Q331K^ (Table 1) mice,^85-87^ while it is decreased upon symptom onset in TDP-43^A315T^ and TDP-43^ΔNLS^ mice.^70,88^ Conversely, IPSC frequency is decreased before symptom onset in SOD1^G93A^, TDP-43^A315T^ and VPS54 loss-of-function mice.^69,89,90^ Comprehensive timeline studies of EPSC and IPSC frequency in layer 5 pyramidal neurons across disease progression in the same mouse model are currently lacking. Ignoring neurotransmission-specific factors, these results can imply that upstream input onto upper motor neurons changes early and skews towards an excitatory imbalance, mirroring findings in patients with amyotrophic lateral sclerosis.^44,91^ It must be noted that EPSC and IPSC measurements via patch-clamp suffer limitations with respect to how they can be interpreted. One issue relates to the physical limitations of clamping neuronal membranes and detecting currents. The amount of control over which experimenters can reliably clamp a membrane, and confidently measure currents, drops as a function of distance from the pipette seal site (known as space clamping issues, Fig. 1). Further, due to separate physical limitations imposed by cable theory, currents that occur at distal sites of axons and dendrites are not resolvable from the soma, which is the most common site where patch-clamp is performed. Another issue with interpreting the frequency of postsynaptic currents is that they are technically the product of two discrete biological processes; notably the physical number of connections formed by presynaptic neurons (neuronal wiring) and the rate of spontaneous neurotransmitter release from presynaptic active zones (synaptic). It is impossible to distinguish which of these two drive changes in the frequency of postsynaptic currents measured from a neuron using whole-cell patch-clamp without supplementary evidence, such as morphological assessment of dendritic spines. The overall result of these limitations is that measurements of EPSCs and IPSCs using patch-clamp likely skew heavily towards axo-somatic connections and cannot readily discriminate between synaptic and wiring defects. One way to overcome these shortcomings is by mapping structural proxies for synapses such as dendritic spines. This approach has been used successfully in rodent models of amyotrophic lateral sclerosis^86,92^ but it is not reliable for silent synapses or inhibitory connections, which are typically aspiny.^93^ The use of membrane- or synaptic-localized calcium indicators to study input at the level of individual synapses and all along the dendritic arbour of upper motor neurons could provide unique insight into how extrinsic excitability changes in amyotrophic lateral sclerosis.

Although the synaptic connections received by upper motor neurons is a useful metric of extrinsic contributions to excitability, it cannot be used as a perfect readout because it does not comment on neuronal activity. It is possible, for example, that increases or decreases in the number of synaptic inputs formed by interneurons onto upper motor neurons are matched by opposite and proportional changes in interneuron activity. In such cases, the extrinsic contribution to upper motor neuron excitability would remain largely unchanged. Interrogation of cortical interneuron excitability, their activity and firing patterns, is a core piece of the puzzle with respect to hyperexcitability in amyotrophic lateral sclerosis. Evidence from TMS clinical and electrophysiological preclinical studies of inhibitory interneurons provides evidence that functional inhibitory drive to upper motor neurons may be pathologically altered. Results from paired-pulse TMS experiments suggest that hyperexcitability of the motor cortex in patients with amyotrophic lateral sclerosis can be ascribed to reduced contribution of cortical inhibitory circuits along with excessive activation of high threshold excitatory pathways.^91,94^ This difference in functional inhibitory-excitatory weighting across the cortex is highly predictive of amyotrophic lateral sclerosis, such that it can differentiate the disease from its mimics^47-50^ and the added presence of increased resting motor threshold can even differentiate amyotrophic lateral sclerosis patients with cognitive impairment from those without.^95^ Evidence from animal models has begun to reveal which specific populations of interneurons are affected in amyotrophic lateral sclerosis. Parvalbumin-positive interneurons, which provide strong direct inhibition to layer 5 pyramidal neurons of the motor cortex, have been found to display increased or decreased excitability in SOD1^G93A^ and TDP-43^A315T^ mice,^66,69,89^ as have GABAergic SOD1^G93A^ cortical neurons *in vitro.*^96^ Somatostatin-positive interneurons, which instead play a more upstream modulatory role via disinhibition, also undergo changes in excitability and notably display a hyperexcitable phenotype in TDP-43^A315T^ mice.^69^ The contribution of these neurons to pathology in amyotrophic lateral sclerosis is highlighted by the finding that ablating them reverses the hyperexcitability phenotype of layer 5 pyramidal neurons.^89^ Cortical excitatory interneurons also display changes that may serve to increase the excitability of upper motor neurons. In particular, corticocortical neurons in the motor cortex of SOD1^G93A^ mice display increased intrinsic excitability.^66^ The relevance of interneurons to amyotrophic lateral sclerosis is highlighted by genome-wide association studies, as well as those employing single cell/nucleus RNA sequencing, which often report that corticomotor system interneurons undergo significant (and usually cell type-specific) transcriptional changes in disease.^97-99^ Although it is evident that upstream changes to upper motor neurons likely play an important role in the pathology of amyotrophic lateral sclerosis, more information is needed to dissect out if these are causal in upper motor neuron pathophysiology. Also, similarly to upper motor neurons, the question of how their activity changes in amyotrophic lateral sclerosis remains unanswered.

### Pre-motor interneurons and spinal cord networks

Although upper motor neurons can form direct connections with lower motor neurons, and thus directly drive glutamate-dependent synaptic transmission, their influence is ultimately dictated by networks of spinal cord interneurons. As such, sources of excitotoxic pressure onto lower motor neurons must always consider the effects of spinal cord network activity and, specifically, the contributions of premotor interneurons. Most studies examining spinal interneurons in patients or preclinical animal models report loss of interneurons as the disease progresses. In some cases, the loss of interneurons precedes loss of lower motor neurons^100,101^ whereas in others, the loss (or magnitude of loss) occurs afterwards^102,103^ or appears to be concomitant.^102,104,105^ This frank loss of interneurons is likely to result in broad changes to the activity of spinal cord networks and, ultimately, lower motor neurons. Consistent with this notion, presynaptic inputs onto lower motor neurons are often impacted in disease. In the SOD1^G93A^ mouse model, lower motor neurons experience generalized loss of presynaptic boutons,^100,106^ indicating that they may become increasingly disconnected from descending and local control. In support of this, many studies have demonstrated that lower motor neurons progressively lose glycinergic connections,^100,101,106^ and that this occurs earliest in the most vulnerable fast fatigable lower motor neurons.^100^ Cholinergic C-boutons, which originate from premotor V0c interneurons, are also lost in SOD1^G93A^ and TDP-43^ΔNLS^ mice,^107-110^ suggesting that affliction of cholinergic input might represent a common pathological outcome of amyotrophic lateral sclerosis. This is consistent with alterations in neurotransmitter release at another cholinergic synapse—the neuromuscular junction.^111-114^ Lastly, studies investigating the recurrent inhibitory circuit mediated by V1 premotor interneurons, known as Renshaw cells, indicate another possible form of disconnect between interneurons and lower motor neurons. Renshaw cell input onto lower motor neurons has been shown to increase at early stages of disease, specifically onto lower motor neurons undergoing degenerative changes.^102,103^ Subsequently, collateral intraspinal axon projections from lower motor neurons onto Renshaw cells retract,^103^ which likely results in a functional decoupling of the recurrent inhibitory circuit. Taken together, these data indicate that spinal cord circuitry undergoes significant remodelling in amyotrophic lateral sclerosis and that the net effect of these changes is a loss of synaptic regulatory control over lower motor neurons.

Assessments of interneuron excitability and activity have been sparse and provide somewhat conflicting reports. Van Zundert *et al.*^115^ examined midbrain interneurons in SOD1^G93A^ neonatal mice (P4–10) and found that they displayed increased excitability and increased frequency of EPSCs and IPSCs. However, a more recent study by Cavarsan *et al*.^116^ reported that glycinergic interneurons in the lumbar spinal cord are hypoexcitable at P6–P10. The most affected glycinergic neurons were those found in the ventral-most domain of the ventral horn (which is largely populated by Renshaw cells), whereas glycinergic neurons in the intermediate region of laminae 7 and 8 were largely unaffected. Because disparate results are found from different regions of the corticomotor system, it is likely that there are interneuron subtype-specific effects on excitability in response to pathology in amyotrophic lateral sclerosis. As such, exploring the neurophysiological profile of different spinal interneuron populations is needed to produce a more complete picture of how spinal network activity is affected in the disease state. Whether this will support or refute the excitotoxicity ‘’dying forward’ hypothesis remains to be seen.

## Synapses

### Basal neurotransmitter release in amyotrophic lateral sclerosis

Core to the ‘dying forward’ excitotoxicity hypothesis is the idea that hyperactivity of upper motor neurons leads to chronic release of glutamate in the spinal cord. A critical question in this hypothesis is: does hyperactivity of upper motor neurons sufficiently produce chronic glutamate release at the synapses in the spinal cord? The neurotransmitter release properties of upper motor neurons have not yet been assessed in the context of amyotrophic lateral sclerosis. This is likely due to the technical challenges associated with accurately measuring basal neurotransmission in the CNS. As such, evidence needs to be extrapolated from existing sources to consider how upper motor neuron synapses might be impacted in disease.

Multiple proteins associated with amyotrophic lateral sclerosis have been shown to localize to presynaptic axon termini,^117-120^ localize to postsynaptic regions^118,120,121^ or directly affect the transcripts of synaptic genes,^119,122-124^ strongly implicating the synapse as a core locus of pathophysiology in the disease.^125^ Assessment of what these changes mean for neurotransmission is challenging but common themes can be extracted. Some RNA sequencing studies indicate that neurotransmission may be impaired/reduced in amyotrophic lateral sclerosis. D’Erchia *et al*.^126^ reported downregulation of over 15 genes associated with synaptic vesicle biology in the spinal cord of patients with amyotrophic lateral sclerosis. These notably included SNAP25 and Syntaxin 1B, which play a critical role in neurotransmitter release. Similar results were found in a study by Liu *et al*.,^98^ which performed single cell RNA sequencing of the SOD1^G93A^ mouse brainstem. A large number of transcripts associated with synaptic transmission, function and assembly were found to be downregulated in symptomatic mice (P100). The most notable drivers of these pathways were decreased expression of neuroligin 1 and neurexin 3, both of which localize to excitatory glutamatergic synapses where they promote synaptic maturation and neurotransmission.^127-129^

Given the many observations of neuronal hyperexcitability in amyotrophic lateral sclerosis, it is possible that reduced synaptic strength occurs as a homeostatic mechanism downstream in the pathological cascade to compensate for increased firing. However, there are many lines of evidence that suggest alterations in basal neurotransmission occur both independently, and in parallel to, synaptic homeostatic responses to changes in neuronal excitability. The first of these is that changes to basal neurotransmission have been observed to occur very early in preclinical amyotrophic lateral sclerosis models^112,130^ and that these changes are also observed *in vitro*^131^ and in development-focused model organisms.^132-134^ The ‘earliness’ of these changes imply a fundamentality to synaptic changes caused by disease-associated mutations. The second is that disease-associated processes are commonly observed to directly interact with or indirectly affect the function of synaptic proteins that are strong determinants of synaptic strength, including synapsin,^131^ SV2,^135^ UNC13A^124^ and GluR1.^136^ The ‘directness’ of these interactions strengthen the fundamentality of the synaptic changes caused by disease-associated mutations. Together, these findings all indicate that neurotransmission in amyotrophic lateral sclerosis may be under constant suppressive pressure in the form of biochemical and molecular pathways that impair neurotransmitter release, and this might play a key role in driving degenerative mechanisms.

In the context of the ‘dying forward’ excitotoxicity hypothesis, these studies recontextualize any observations of increased upper motor neuron or premotor interneuron activity. This is because if these excitatory neurons suffer from suppressed neurotransmitter release, any increases in their activity might only serve to normalie glutamate release over time. It must be noted that changes in glutamate handling extrasynaptically have been postulated to occur in amyotrophic lateral sclerosis due to changes in the glutamate transporter, EAAT2. Decreased expression of EAAT2 is strongly correlated with degeneration in the ventral spinal cord of deceased amyotrophic lateral sclerosis patients^137^ and patient-derived synaptosomes are less competent at transporting glutamate.^138^ EAAT2 expression is also decreased in rodent models of amyotrophic lateral sclerosis^139,140^ as well as in dogs with canine degenerative myelopathy, which is a naturally occurring model of motor neuron disease.^141,142^ Together, these suggest that reduced EAAT2 may represent a strongly conserved pathological signature of amyotrophic lateral sclerosis. How this fits into our picture of pathogenesis remains to be determined as clinical trials targeting EAAT2 have proved ineffective.^11^ However, changes in glutamate handling must always be considered in the context of neuronal activity over time because activity-dependent synaptic vesicle release is a major source of extracellular glutamate. One possibility that has received little appreciation is that the low glutamate concentrations in the ventral horn of amyotrophic lateral sclerosis patients’ spinal cords might suggest that the combined changes in neuronal activation and glutamate handling results in a net reduction. More work on deciphering the sources and sinks of glutamate in the motor cortex and spinal cord will be needed to resolve this question. For example, a key missing piece of evidence for the excitotoxicity ‘dying forward’ hypothesis, and our understanding of the corticomotor system in general, are the neurotransmission properties of upper motor neuron axon terminations in the spinal cord (Fig. 1). Do they release more, or less, glutamate per action potential or over time? Although technically challenging, exploring upper motor neuron synaptic output will undoubtedly provide a core piece of evidence towards our understanding of the ‘dying forward’ hypothesis, and thereby the role of glutamate-mediated excitotoxicity.

## Conclusion and key messages

The past three decades of attempting to treat amyotrophic lateral sclerosis by suppressing neuronal activation across the whole nervous system has demonstrated that our theories of the disease need to be revisited. The ‘dying forward’ hypothesis remains an attractive explanation for pathogenesis in amyotrophic lateral sclerosis. In support of this, studies on ALS patients and preclinical animal models converge on the concept that the motor cortex and the upper motor neurons display hallmarks of increased excitability either in the early stages of ALS or in the presymptomatic phase of familial ALS.^44^ While motor cortex hyperexcitability is clearly associated with amyotrophic lateral sclerosis, the precise molecular processes underlying excitotoxicity warrants further assessment. Mechanisms responding to potential perturbations in excitability need to be differentiated from pathological drivers of amyotrophic lateral sclerosis at a neuronal network, cellular and synaptic level along the corticospinal tract (Fig. 1). Fortunately, there are no technological limitations to our ability to ask these questions with the flurry of diverse and spatiotemporally accurate tools that the past decades have provided. Hopefully, recognition of this promotes increased focus on research into upper motor neuron activity *in vivo* as well as assessments of synaptic biology in amyotrophic lateral sclerosis.
